# Layer number identification of CVD-grown multilayer graphene using Si peak analysis

**DOI:** 10.1038/s41598-017-19084-1

**Published:** 2018-01-12

**Authors:** You-Shin No, Hong Kyw Choi, Jin-Soo Kim, Hakseong Kim, Young-Jun Yu, Choon-Gi Choi, Jin Sik Choi

**Affiliations:** 1 Department of Physics, Konkuk University, Seoul, 05029 Korea; 2 Emerging Devices Research Group, Electronics and Telecommunications Research Institute (ETRI), Daejeon, 34129 Korea; 3 Department of Physics, Korea University, Seoul, 02841 Korea; 4 Korea Research Institute of Standards and Science (KRISS), Daejeon, 34113 Korea; 5 Department of Physics, Chungnam National University, Daejeon, 34134 Korea; 6 Graphene Research Lab., Emerging Devices Research Group, Electronics and Telecommunications Research Institute (ETRI), Daejeon, 34129 Korea

## Abstract

Since the successful exfoliation of graphene, various methodologies have been developed to identify the number of layers of exfoliated graphene. The optical contrast, Raman G-peak intensity, and 2D-peak line-shape are currently widely used as the first level of inspection for graphene samples. Although the combination analysis of G- and 2D-peaks is powerful for exfoliated graphene samples, its use is limited in chemical vapor deposition (CVD)-grown graphene because CVD-grown graphene consists of various domains with randomly rotated crystallographic axes between layers, which makes the G- and 2D-peaks analysis difficult for use in number identification. We report herein that the Raman Si-peak intensity can be a universal measure for the number identification of multilayered graphene. We synthesized a few-layered graphene via the CVD method and performed Raman spectroscopy. Moreover, we measured the Si-peak intensities from various individual graphene domains and correlated them with the corresponding layer numbers. We then compared the normalized Si-peak intensity of the CVD-grown multilayer graphene with the exfoliated multilayer graphene as a reference and successfully identified the layer number of the CVD-grown graphene. We believe that this Si-peak analysis can be further applied to various 2-dimensional (2D) materials prepared by both exfoliation and chemical growth.

## Introduction

Graphene has attracted great interest from researchers in various fields since the first successful separation of monolayer graphene from graphite^1^. Single-layer graphene is a promising material for nanoelectronics because of its high electrical transport properties that can be controlled by an applied electric field^2^. The high optical transparency^3^, chemical stability^4^, and high mechanical strength^5^ render single-layer graphene versatile for nanophotonic and optoelectronic applications and flexible electronics. The importance of multilayer graphene (MLG) has also rapidly grown for the past few years because of its unique functionality and wide applicability in various optical and electrical nanodevices. Gate-tunable bandgaps particularly enable the fundamental investigation of graphene-based optoelectronics^6–8^. In addition, multilayer graphene offers an unprecedented opportunity to flexible electronics, thereby providing optically transparent, atomically thin, and ultra-flexible electrical contacts (e.g., electrical contacts for two-dimensional (2D) van der Waals heterostructures (vdWs))^9^. The most remarkable feature of these layered 2D materials including graphene is the layer-number-dependence of the physical properties even in the same material. Therefore, the precise control of the number of layers in 2D materials has become one of the most critical steps in fabricating 2D nanodevices. In this regard, controlling the layer numbers of 2D van der Waals materials with an unambiguous and reliable identification method is required first.

The optical contrast technique is a rapid and easy method of confirming the layer number of multilayer graphene representing the sample thickness by the contrast of reflection spectra or color difference. However, the technique is only useful in relative thickness comparison, and has a limited range that can be measured depending on the SiO_2_ thickness^10,11^. Other optical techniques have been introduced to identify the number of layers of multilayer graphene, including Raman spectroscopy^12–17^ and Rayleigh scattering^18^. Raman spectroscopy is more widely used because it not only provides more information about the layer number of a given sample, but also reveals important physical properties, such as phonons, electron–phonon coupling, band structures, and interlayer coupling of multilayer graphene. The most discernable two peaks in the Raman spectrum of graphene and graphite are the G-peak (1580 cm^−1^) and the 2D-peak (~2700 cm^−1^). For exfoliated graphene the number of layers can be reliably determined from the line shape of the 2D peak for fewer than 7 or 8 layers^12–14^. The intensity ratios of G/2D^15^ and G/Si^16^ are also introduced to facilitate the counting process using the Raman G- or 2D-peak. However, they are reliable only in limited ranges. For example, the G-peak intensity of a thin-layer region tends to increase with the function of the layer number, but decrease in the thick layer region (>18 layers)^17^. Moreover, the 2D-peak of the multilayer graphene is composed of multiple peaks. Therefore, the countable number is limited for thin layers. Xiao-Li *et al*. recently reported the layer number identification of exfoliated multilayer graphene with a wide countable range (1–100 layers) using the Si substrate peak intensity^17^. They used multiple reflection interference method of constitution of air, multilayer graphene, SiO_2_, Si with complex refractive index and the thickness of each medium. No reversible point limits the identification range in the case of the Si-peak intensity. Moreover, 532 nm excitation gives a good optional choice for layer number determination for various SiO_2_ layer thicknesses (285 nm < ${h}_{{{\rm{SiO}}}_{2}}$ < 305 nm or 90 nm < ${h}_{{{\rm{SiO}}}_{2}}$ < 110 nm) since it does not show the thickness dependence. In their work, the reliability of the Si-peak intensity was verified with various experimental parameters, such as the SiO_2_ layer thickness, laser excitation wavelength, and numerical aperture of the objective. This method was also used for counting the number of layers of other exfoliated 2D materials^19,20^. However, despite various attempts at exfoliated graphenes, no reliable technique has been proposed to clearly identify the number of layers of chemical vapor-deposited (CVD) graphene. For CVD-graphene, since the 2D-peak line-shape changes significantly by the twisted angles between the layers even on the same layer numbers, the number identification becomes quite complicated than the exfoliated graphene. The verifying number of randomly stacked large-scale multilayer graphene accompanied with a twisted angle is an essential step for transparent and flexible electronic applications. Hence, a credible avenue capable of identifying the number of graphene is demanded.

In this work, we identified the usefulness of the Si-peak analysis in determining the number of layers of CVD-grown graphene using 532 nm excitation. We compared the optical characteristics between an optical image and the Raman images of the Si-, G-, and 2D-peaks to determine the number of layers of graphene grown by CVD. We confirmed that the CVD-grown graphene has many disorders because of the twisted angle between the layers, which caused 2D- and G-band variations even at the same layer number^21–25^. Only the Si-peak exhibited a gradual intensity variation as the number of layers increased or decreased. In addition, the peak positions remained unchanged for all of the graphene layers. This comparison confirmed that the Si-peak analysis was robust, and a powerful characterization tool for identifying the number of layers in CVD-grown multilayer graphene.

## Results

**Figure 1:**
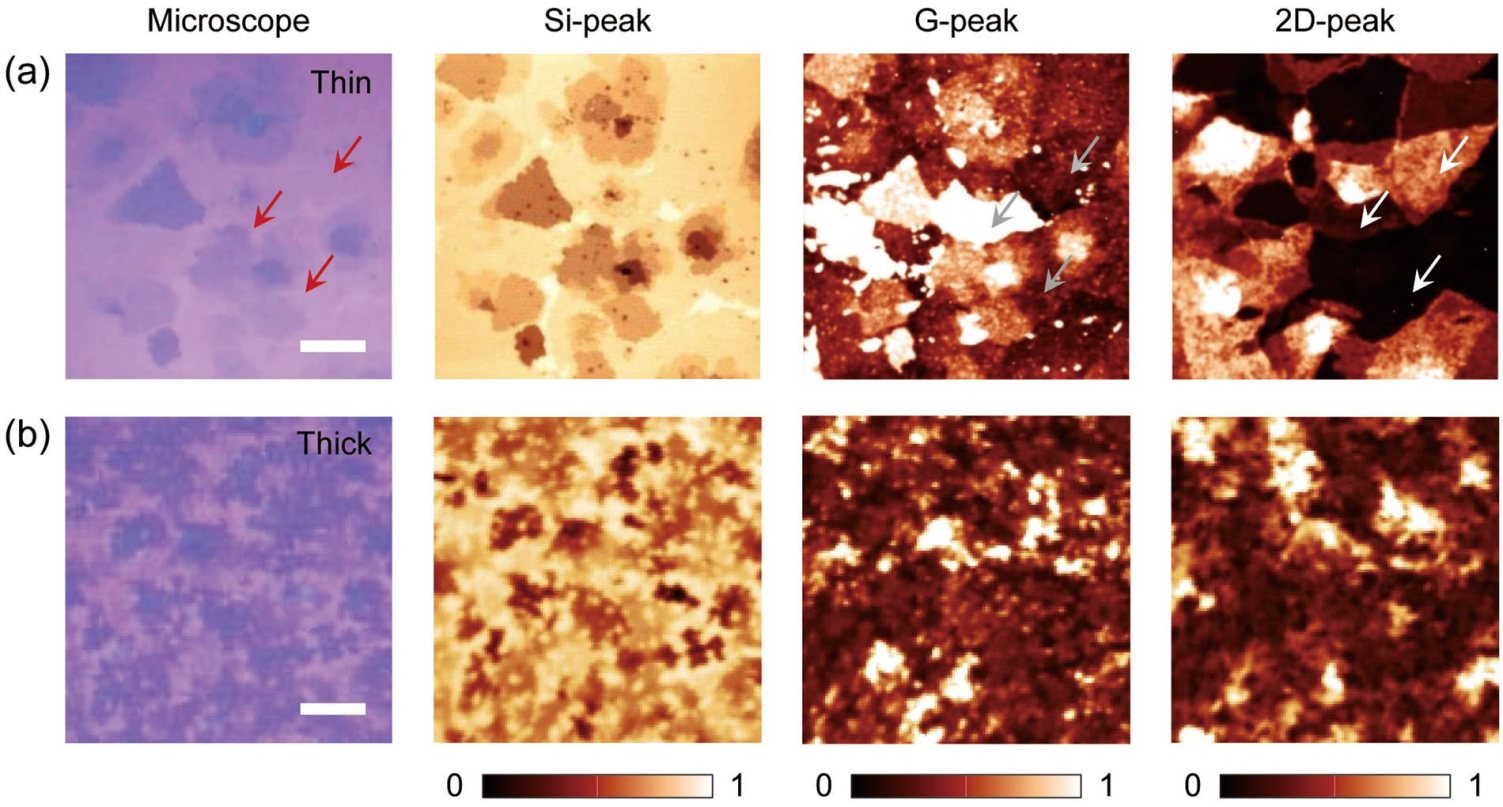
Optical microscopy and Raman spectroscopy images of the CVD-grown graphene. The optical images (first column) and various Raman mapping images of the integrated intensities of the Si- (450–600 cm^−1^, second column), G- (1490–1670 cm^−1^, third column), and 2D- (2600–2780 cm^−1^, fourth column) peak bands of the CVD-grown with (**a**) thin and (**b**) thick multilayer graphene. In all Raman spectroscopy images, an incident laser with an excitation wavelength of 532 nm was used. The scale bars in (**a**) and (**b**) are 20 and 10 μm, respectively. Each Raman mapping image was normalized with its maximum value.

Figure 1(a) shows the representative optical microscopy and various Raman spectroscopy images of CVD-grown multilayer graphene consisting of a variety of sub-domains of thin graphene layers. The optical image [first column, Fig. 1(a)] exhibits a weak optical contrast between the graphene layers with different layer numbers, which made it difficult to identify the boundaries of the individual graphene domains and the layer number distributions. In Raman spectroscopy, the intensity mappings of the G-peak and 2D-peak [third and fourth columns, Fig. 1(a)] show different spatial distributions of peak intensity. For example, the regions with a similar optical contrast in the microscopy image (red arrows in the first column), had different intensity variations in the G-peak (gray arrows in third column) and 2D-peak (white arrows in fourth column) mapping images. In addition, it was even more difficult to match the boundaries of the graphene domains and the corresponding layer numbers in an optical microscopy image with those in the G- or 2D-peak mapping images. These results reveal that our CVD-grown large area graphene was composed of various local domains with different crystal orientations^22–25^. Consequently, without knowing the exact layer number and layer boundaries, further material characterizations and analyses of CVD-grown multilayered graphene are not possible. However, the Si-peak intensity mapping image [second column, Fig. 1(a)], provides a much clearer and improved visualization of the graphene boundaries and layer number information with better resolution. For example, the intensity mapping revealed not only the clean edges of the individual graphene domains but also many small dots of thin graphene, which were hardly obtainable from the optical microscopy image.

The Si-peak intensity mapping has more advantages in analyzing thick multilayered graphene with small domains. We synthesized thick multilayered graphene via the CVD method and performed Raman spectroscopy to obtain the intensity mapping images [Fig. 1(b)]. In the optical microscope images [first column, Fig. 1(b)], it was more challenging to identify the detailed spatial distribution of the graphene layer number because of large thickness variations and small domain sizes. In addition, the G- and 2D-peak intensity mappings reveal more complex peak intensity variations. In particular, the comparison of the G- and 2D-peak mappings showed many discrepancies between the locations and peak intensities. As a result, these cannot be compared using the optical microscope images for determining the layer number distribution. Conversely, the Si-peak intensity mapping showed relatively clear boundaries and intensity contrast between the multilayered graphene with small domain sizes. This consequently revealed the spatial distribution of the graphene layer number with better resolution, which also agreed well with the optical contrast in the optical microscope image.

**Figure 2:**
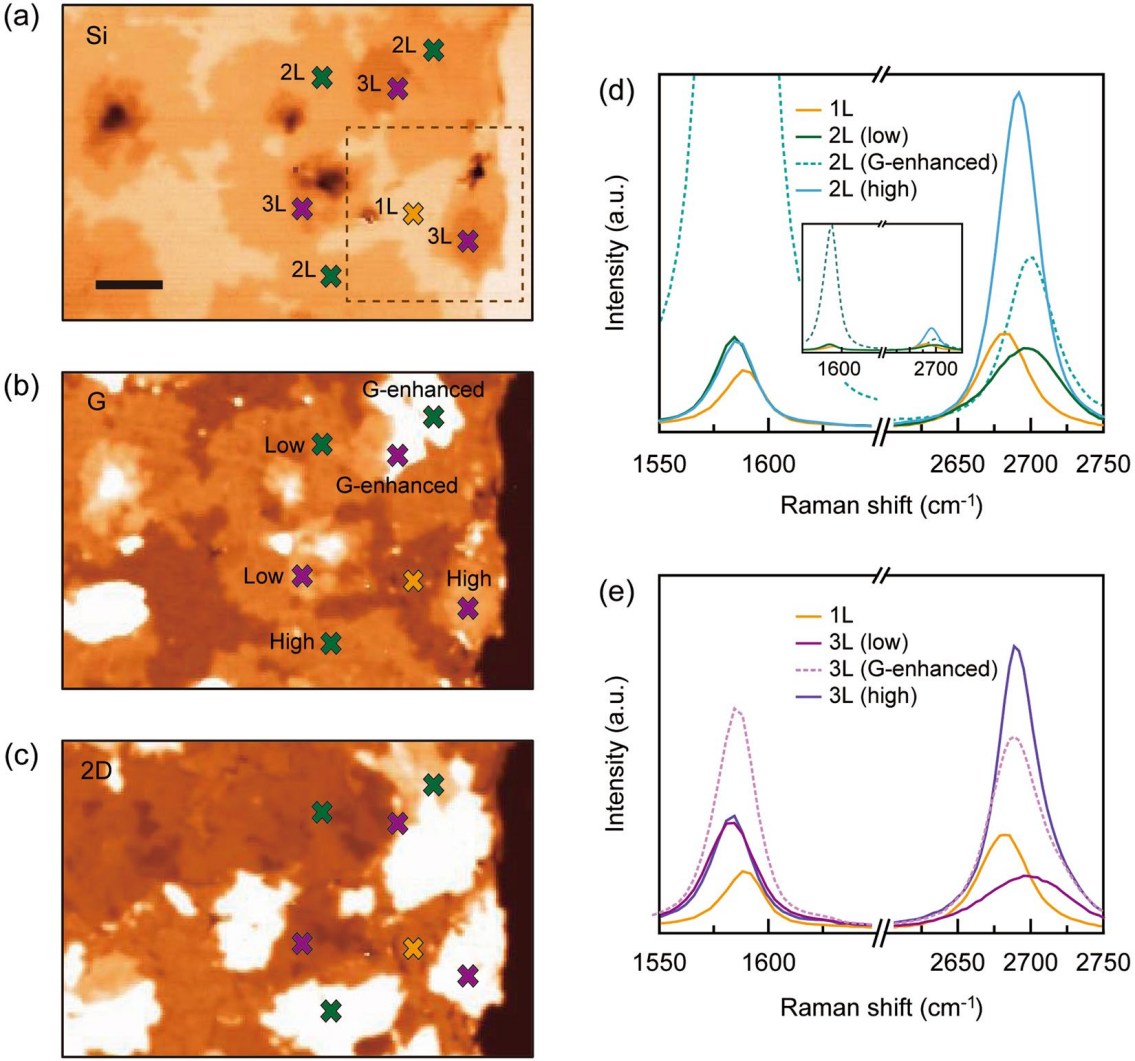
Spectroscopic analysis of Raman intensity mapping in CVD-grown graphene on a SiO_2_/Si substrate. The Raman intensity mapping images of (**a**) Si-, (**b**) G-, and (**c**) 2D-peaks measured from the CVD-grown graphene with an area of 70 × 40 μm^2^. The thickness of SiO_2_ was 300 nm. The scale bar is 10 μm. (**d**,**e**) Separately obtained Raman spectra of the G- (~1580 cm^−1^) and 2D-peaks (~2700 cm^−1^) at different positions in the bi- [2 L, (**d**)] and tri- [3 L, (**e**)] layers. The Raman spectrum of single-layer graphene was co-plotted for reference. In each plot, the labels “low” (dark-straight line), “G-enhanced” (dotted line), and “high” (bright-straight line) indicate the twist angles between the two misoriented layers, corresponding to low-, specific middle- and high-angles, respectively. They were sorted by the characteristics of the G- and 2D-peak intensities and shapes^21^. The denotations of colors in (**d**) and (**e**) are the same as those in (**a**–**c**).

For a systematic study, we synthesized a thin multilayered graphene sample with small spatial variations in layer number on a SiO_2_ substrate. We performed Raman spectroscopy and obtained the Raman Si-, G-, and 2D-peak intensity mapping images in an area of 70 × 40 μm^2^, [Fig. 2(a–c), respectively]. The Si-peak intensity mapping image showed a gradual increase in the peak intensity between individual graphene domains that were distributed on the SiO_2_/Si substrate. The dark colored regions represent the thick multilayered graphene, which acted as the seed layer^26^. Before we analyzed the detailed spectroscopic features of the Si-peak with increasing layer number, the layer numbers of the individual graphene domains had to be identified and matched with the corresponding Si-peak intensities. The single-layer graphene (1 L) region was readily identified with the highest intensity in the Si-peak mapping image and a unique local spectrum of single Lorentzian G- and 2D-peaks, as well as the uniform intensity measured by the optical microscope, in the G- and 2D-peak mapping images. However, the subsequent process was not straightforward because the major criteria for layer number identification used in exfoliated multilayer graphene are not suitable for CVD-grown multilayer graphene^22–25^.

A critical factor that produces the dominant differences in the Raman characteristics is the angle between layers. For example, the Raman spectroscopy of exfoliated graphene always exhibits the same tendency with increasing number of graphene layers, as the crystallographic axes between layers are perfectly aligned. Conversely, CVD-grown graphene consists of various domains with randomly rotated crystallographic axes between layers. Consequently, the interlayer angles cause distinctive features in the Raman spectra, which are not found in exfoliated graphene. In this regard, we carefully chose several locations in the Si-peak mapping and categorized them according to their intensity: the green and violet markers for the second and third highest intensity values [Fig. 2(a)]. In addition, we obtained G- and 2D-peak Raman spectra from these locations [the same colored markers in Fig. 2(b),(c)] and analyzed the results. In Fig. 2(d), we plotted the measured spectra from the locations with green colored markers. There were several distinctive spectral features. First, we observed a strongly enhanced G-peak spectrum from one location (the green marker labeled “G-enhanced” in Fig. 2(b) and the dotted line in the inset of Fig. 2(d)], whereas the other two locations showed negligible spectral differences in either intensity or profile. Secondly, all three G-peak intensities were higher than that of single-layer graphene (yellow line). Thirdly, different intensities and line-shapes of the 2D-peaks were observed. In particular, one of them (dark green line) showed a lowered intensity and broadened line-shape compared with those for the single-layer graphene.

We note that all these observations represent the characteristic spectral features originating from the various interlayer angles in multilayered graphene^21^. For example, the low angle twisted bi-layer graphene exhibits a much broader and upshifted 2D-band. The high rotation angle yields a single Lorentzian line-shape similar to that of a monolayer graphene and the peak intensity increased as the rotation angle increased. For the G-band, there is a pronounced enhancement in the spectrum for a specific angle and incident laser excitation^21,22,25^. Therefore, based on our observations and analyses, we labeled the three graphene domains with green colored markers in Fig. 2(a–c) as “low”, “G-enhanced”, and “high”, which corresponded to low, intermediate, and high twisted angles, respectively. Furthermore, we also used the same procedure on the violet colored markers and labeled these “low”, “G-enhanced”, and “high” in Fig. 2(a–c), respectively. Lastly, we directly compared the intensities of the Si-, G- and 2D-peaks with low angles [dark-straight lines in Fig. 2(d),(e)] as well as the optical contrasts in the microscope images and identified the layer numbers of the domains where the green (violet) colored markers were located as bilayer (trilayer) graphene. Although we have successfully identified single-, bi-, and tri-layer graphene, the Raman spectroscopic analysis based on the G- and 2D-peaks revealed critical limitations; the angle-dependent and irregular peak intensity variations. As a result, an unambiguous identification of layer number is complicated when the number of layers is greater than 3, providing strong motivation to find a simple and robust way to determine the layer number in CVD-grown multilayer graphene.

**Figure 3:**
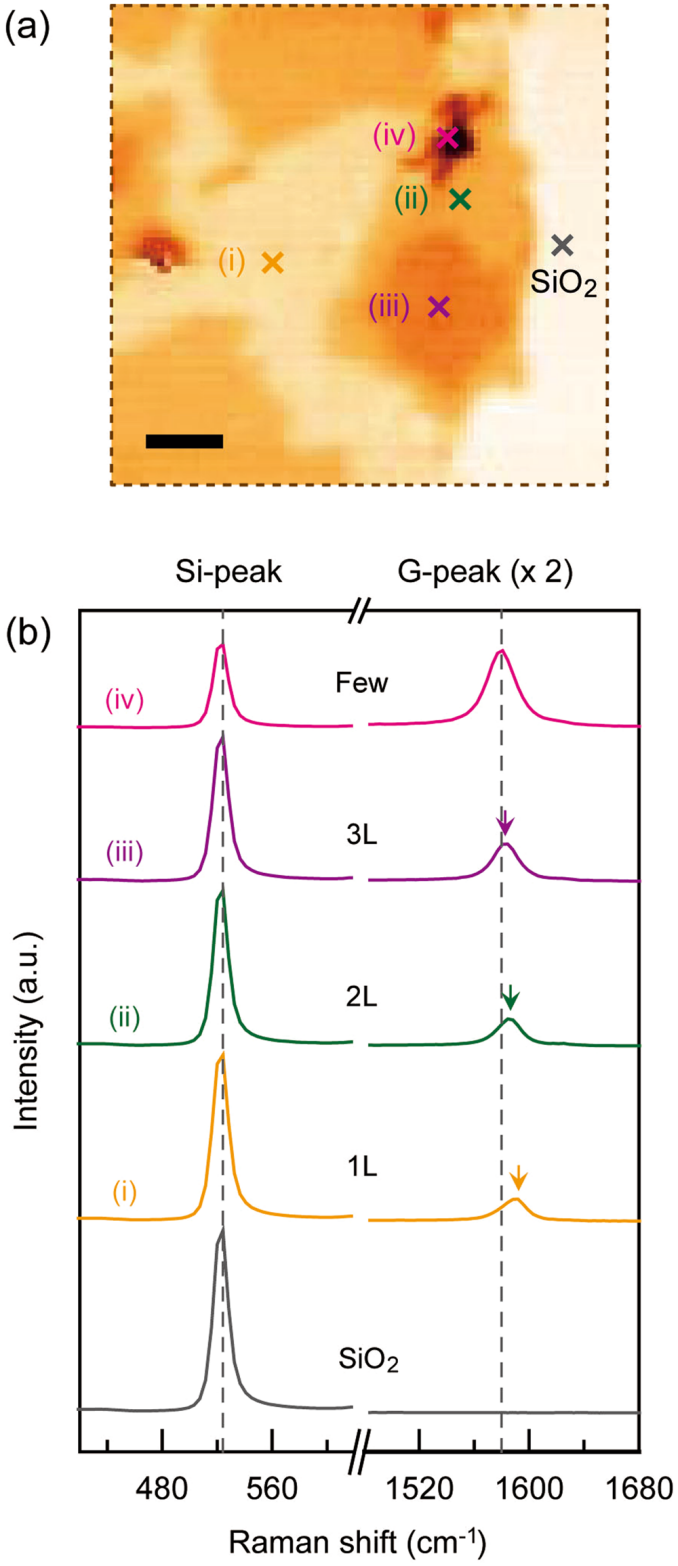
Raman intensity analysis of the Si- and G-peaks as a function of number of layers in CVD-grown graphene. (**a**) Magnified Raman mapping image of the selected area: 30 × 30 μm^2^ (brown dotted line) in Fig. 2(a). The scale bar is 5 μm. (**b**) Raman spectra of the Si- (~520 cm^−1^) and G- (~1580 cm^−1^) peaks at the positions of (**i**)-(iv) and SiO_2_ in (**a**). Here, the intensities of the G-peak spectra were doubled for a better comparison. The colored arrows indicate the maximum G-peak positions in the individual layered graphene samples. The denotations of colors in (**a**) and (**b**) are the same as those in Fig. 2(a)–(c). The pink color was used for the few layer graphene.

Figure 3(a) shows a magnified Si-peak intensity mapping image of the selected area in Fig. 2(a). We observed a gradual peak intensity variation, which reveals there are successive layers of graphene. We chose several locations with different peak intensities (colored markers) and performed Raman spectroscopy. Figure 3(b) shows the Raman spectra of the Si- and G-peaks from single- [yellow, (i)] to a few- [pink, (iv)] layered graphene. Here, we selected the Raman spectra of the twist angled samples that had characteristic features of low- or high-rotation angles. The Si-peak revealed a gradual decrease in intensity with an increasing number of layers of graphene, while the maximum peak positions (~520 cm^−1^) and overall line-shapes (FWHM ~28 cm^−1^) remained unchanged. This monotonic variation in Si-peak intensity arises from the absorption of both the excitation power and Si Raman signals on the graphene samples, which consequently enabled the identification of the number of layers. In addition, we did not observe any angle-dependent spectral features from the Raman spectra of the Si-peak, which suggest that the Si-peak is more suitable as an effective tool in identifying the number of layers of multilayer graphene with various rotation angles between layers. Conversely, the Raman G-peak spectrum clearly exhibited irregular variations in line-shape and peak position as the number of layers increased from single to a few, although it showed a gradual increase in intensity.

**Figure 4:**
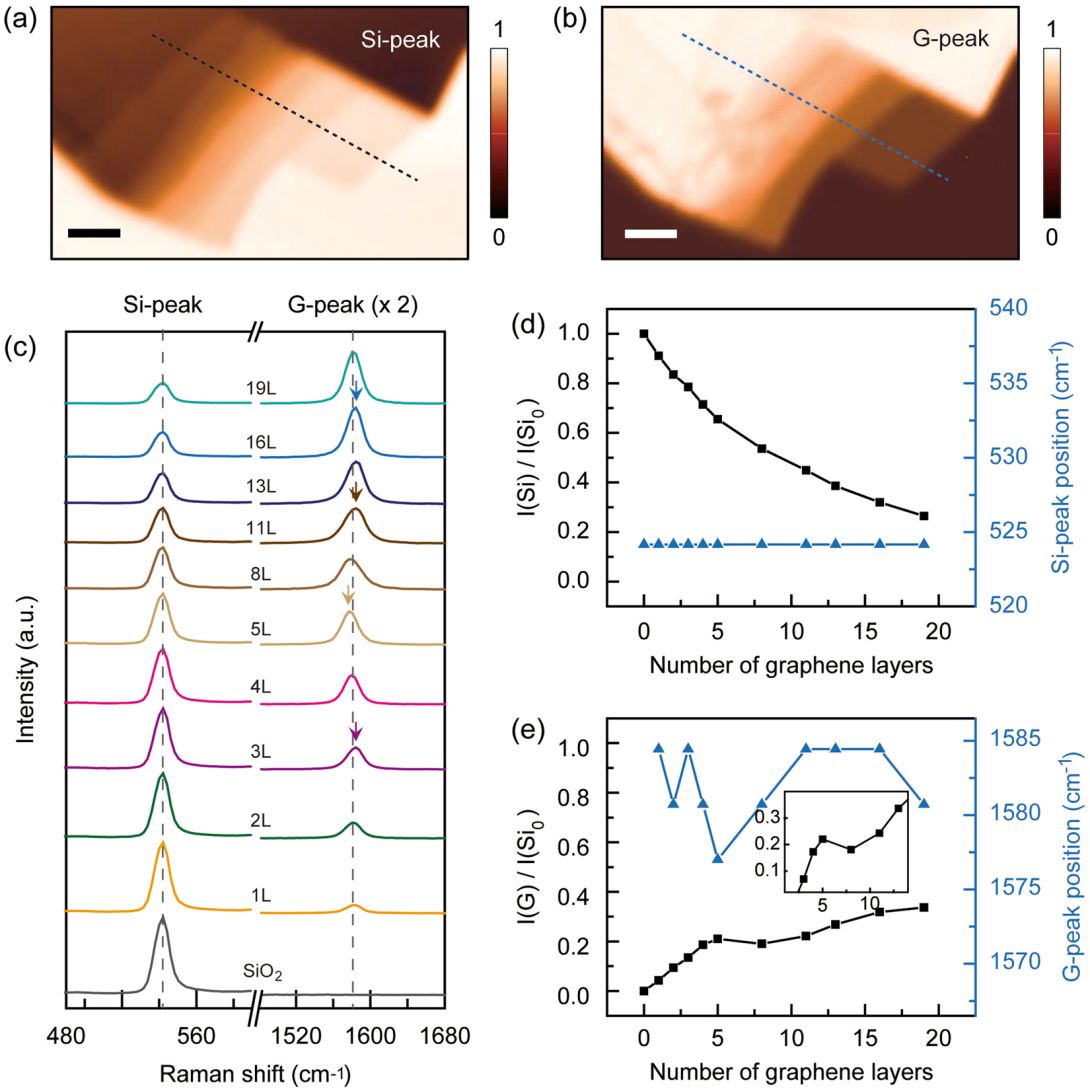
Raman intensity mapping analysis of exfoliated multilayer graphene. (**a**,**b**) Raman intensity mapping images of the (**a**) Si- and (**b**) G- peaks; the scale bars are 5 μm. (**c**) Various Raman spectra of the Si- (~520 cm^−1^) and G- (~1580 cm^−1^) peaks depending on the layer numbers and SiO_2_. The colored arrows indicate the maximum peak positions of the layered graphene. (**d**) Co-plotted Si-peak intensity [*I*(Si), black squares] and corresponding peak position (blue triangles) as a function of the number of layers. (**e**) Co-plotted G-peak intensity [*I*(G), black squares] and corresponding peak position (blue triangles) as a function of the number of layers. All Si-peak and G-peak intensities were normalized with *I*(Si_0_). Here, *I*(Si_0_) is the Si-peak intensity of the SiO_2_/Si substrate (0 L). Inset: a local turning point of *I*(G)/*I*(Si_0_) with increasing layer number.

To examine the validity of the Raman Si-peak intensity as a universal measure for the number identification of multilayered graphene, we separately prepared a mechanically exfoliated graphene sample and repeated the systematic Raman spectroscopy. Figure 4(a,b) show the Raman mappings of the Si- and G-peak intensities, respectively. For a direct comparison, we prepared the sample with a continuous increase of layer number in the thin region [1L–5 L, bottom right in Fig. 4(a,b)] and a gradual variation with 2 or 3 intervals in the thick region (8L–19 L, top left in Fig. 4(a,b)]. We used atomic force microscopy, confirming the number of layers and the topographical distribution (See Supplementary Information S1). In Fig. 4(c), we show the Raman spectra of the Si- and G-peaks obtained from every graphene layer in Fig. 4(a,b). We observed a gradual intensity variation of the Si-peak as the number of layers increased or decreased. The peak positions remained unchanged for all of the graphene layers. However, the G-peak intensity showed a different tendency. It initially increased in the thin graphene layer region (<8 layers) as the number of layers increased. Then it revealed an intensity local minimum, showing the decrease and increase of intensity as the layer number was increased. We also observed spectral shifts in the G-peak when varying the layer number (colored arrows), but no noticeable tendency was observed. As shown in previous studies of exfoliated multilayer graphene, the G-peaks tend to increase with the number of layers in the thin layer region (<18 layers), then decrease as the number of layers increases (>18 layers)^13,17^. We have confirmed this reduction in the G-peak intensity of exfoliated multilayer graphene in the thicker layer region from another sample, while observing that the Si-peaks monotonously decreased in this region (See Supplementary Information S2).

In Fig. 4(d,e), we quantified and summarized the results of Fig. 4(c). We first normalized all of the Si-peak intensities [*I*(Si)] with respect to that of the SiO_2_/Si substrate [*I*(Si_0_)]. Then we co-plotted *I*(Si) and the corresponding peak positions as a function of the number of graphene layers [Fig. 4(d)]. The result clearly revealed a gradual decrease of the normalized intensity of the Si-peak with increasing layer number. In addition, the maximum peak position (~520 cm^−1^) and the linewidth (FWHM ~19.1 ± 0.3 cm^−1^) of the Raman spectra of the Si-peak remained unchanged. All these tendencies agree well with the observations from the CVD-grown multilayered graphene sample in Fig. 3, which suggests that the Raman Si-peak can be used as an efficient, robust, and universal measure for identifying the number of layers in multilayered graphene. Next, we also normalized the G-peak intensities [*I*(G)] with respect to *I*(Si_0_) and co-plotted them with the corresponding peak positions as a function of the number of graphene layers [Fig. 4(e)]. However, the results exhibited a complicated behavior of the G-peak. For example, we observed intensity turning points (inset) as the layer number increased. These turning points were also confirmed in the continuous line profile of the G-mapping image, but the topographical height and Si-peak intensity were still in the increasing region. Although this intensity behavior in the thin layer region (<18 layers) was not observed, an intensity complexity exists in a similar region in a previous report^17^. We also observed that the maximum peak position of the G-peak irregularly shifted within the range of 10 cm^−1^. Combining these observations, all features of the Raman Si- and G-peaks are consistent with the observations in the CVD-grown graphene in Fig. 3, confirming that the Raman Si-peak is more suitable than the Raman G-peak for layer number identification.

**Figure 5:**
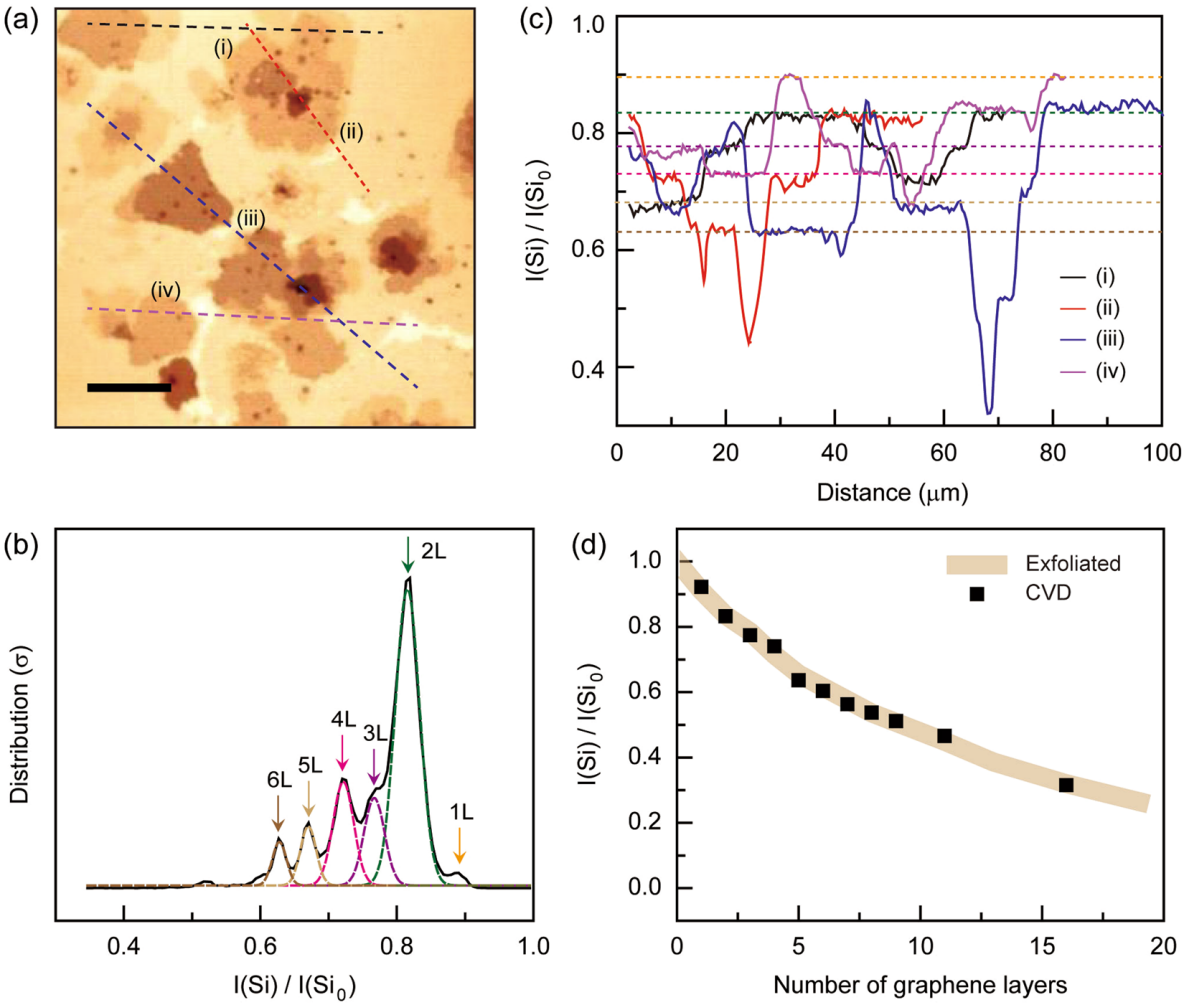
Layer number identification of CVD-grown graphene based on the Raman intensity of the Si-peak. (**a**) Raman intensity mapping of the Si-peak measured from the CVD-grown thin multilayer graphene on SiO_2_/Si substrate [second column in Fig. 1(a)]. The mapping area was 100 × 100 μm^2^. The scale bar is 20 μm. (**b**) The Si-peak intensity distribution of various multilayered graphene samples extracted from the intensity mapping image of (**a**). Multiple Gaussian curves with various colors were used to fit the distribution peaks from 2 L to 6 L. (**c**) Four representative intensity line profiles along the (i) black, (ii) red, (iii) blue and (iv) purple lines in (**a**). Each line profile was plotted from left to right along the respective line. The colored dotted guidelines correspond to the Si-peak intensities of the 1 L to 6 L graphene samples, from top to bottom. The denotations of colors in (**c**) are the same as those in (**b**). (**d**) The number identification of the CVD-grown multilayer graphene. The normalized Si-peak intensities [*I*(Si)/*I*(Si_0_)] from the exfoliated multilayer graphene in Fig. 4 are plotted as a function of the number of layers, and used for reference (thick brown colored line). The *I*(Si)/*I*(Si_0_) data from the CVD-grown multilayer graphene in (**a**) are co-plotted.

In Fig. 5, we explore the applicability of the Si-peak intensity as an efficient measure to identify the number of layers (>3 L) in CVD-grown multilayered graphene by using the measured data in Fig. 4. First, we chose the Raman mapping image of Si-peak intensity from Fig. 1(a) [Fig. 5(a)] and performed a systematic layer number analysis [Fig. 5(b)–(d)]. We separated the individual peaks from the peak intensity mapping and re-plotted them as a function of intensity in Fig. 5(b). In the intensity distribution plot, we used multiple Gaussian fitting curves to fit the intensity distribution data. We also separately performed Raman spectroscopy and obtained Si-peak intensity values of 1 L to 3 L graphene. By correlating these values, we successfully confirmed single-, bi-, and tri- layer graphene in Fig. 5(a). We were able to distinguish the intensity signal of the single-layer from the distribution although the fitting curves were limited for single- and thicker layers (>6 L) due to insufficient data collected from the small areas. In addition, the other multilayered graphene samples (4L–6 L) were identified by the Gaussian fitting curves of intensity distribution. All of the Si-peak intensities of the graphene layers [*I*(Si)] were normalized with the Si-peak intensity of the SiO_2_/Si substrate [*I*(Si_0_)].

Figure 5(c) exhibits the four representative intensity line profiles plotted as a function of scanning distance. In the Si-peak intensity mapping, we selectively line-scanned along the regions where the Si-peak intensities differed. The (i) black, (ii) red, (iii) blue, and (iv) purple intensity profiles correspond to the respective lines with the same colors in Fig. 5(a). In each intensity line plot, we clearly observed several local intensity plateaus. For example, the purple colored intensity line plot [the indicated line-scan (iv) in Fig. 5(a) and (c)] shows the first local plateau (1 L) in the scan distance range of 30–33 μm, second plateau (2 L) in the range 61–74 μm, and third plateau (3 L) in the range 5–15 μm, with all other cases (4L–6 L) also shown from top to bottom. Along the line profile, the layers are distributed in the order of 3L–4L–1L–3L–4L–3L–5L–2L–1 L, from left to right. Other intensity line plots [(i)-(iv)] show several plateaus in various scan ranges and some of these exhibit the same intensity values, which indicate that the line scans passed through the graphene with the same layer number. Notably, we observed that the intensity plateaus are exactly matched with those from the levels of the Si-peak intensities of 2 L (green arrow), 3 L (purple arrow), 4 L (cyan arrow), 5 L (yellow), and 6 L (brown arrow) in Fig. 5(b), which re-confirmed the previous results and analysis.

Finally, we used the normalized Si-peak intensity data measured from the exfoliated multilayer graphene (Fig. 4) as a reference to determine the layer number of CVD-grown multilayer graphene. In Fig. 5(d), we co-plotted *I*(Si)/*I*(Si_0_) from both the exfoliated (thick brown line) and CVD multilayer graphene (black squares). By directly comparing these values, the layer numbers of the thick multilayer CVD graphene (9 L, 11 L, and 16 L) were successfully determined. We note that there are several advantages in using this Si-peak for identifying the layer number in CVD graphene. First, the described method is simple and only requires Si-peak intensity data. In addition to the layer number identification, the Raman mapping results can be used to obtain the local information of the D-, G-, and 2D-peaks for further analysis, such as structural disorder and electrical doping. Second, the described quantity of normalized I(Si) [*I*(Si)/*I*(Si_0_)] can be universally applied to identify the number of layers for both exfoliated and CVD graphene, which reveals that the Si-peak is not significantly affected by undesirable experimental factors such as PMMA residues and structural disorders commonly observed in CVD-grown multilayer graphene. Third, the Si-peak shows no angle dependency in twisted multilayer graphene, which is a key limitation of other Raman spectroscopic methods.

## Conclusion

We demonstrated a reliable identification of the number of layers of CVD-grown multilayer graphene. We obtained the normalized Si-peak intensities of exfoliated multilayer graphene, and applied it to CVD-grown multilayer graphene. CVD-grown multilayer graphene has complex G- and 2D-band variations depending on the crystal disorder even in the same number of layers. However, using normalized Si-peak intensities to identify the number of graphene layers was useful for both CVD-grown and exfoliated multilayer graphene. We believe that the Si-peak analysis is the most powerful tool for determining the number of layers of multilayer graphene samples although the further study for different types of substrates would be necessary. Furthermore, this identification method can be exploited for Van der Waals heterostructures made of various 2D materials such as hBN and TMDCs, when substrate related peaks are found and the relationship with the number of layers is verified.

## Methods

### Synthesis of Graphene

The multilayer graphene samples were synthesized using a Cu/Ni metal catalyst by chemical vapor deposition^27^. The thick multilayer graphene was synthesized using a 300 nm Ni metal catalyst, and the thin multilayer was grown on a Cu(400 nm)/Ni(300 nm) metal catalyst. The metal catalyst films were deposited onto a thermal oxidized 300 nm thick SiO_2_/Si substrate, which was then heated up to 1,000 °C inside a CVD under H_2_ atmosphere, and the graphene was then grown with flowing gas mixtures of H_2_:CH_4_ = 10:5 (sccm) for 20 min. After synthesizing the graphene, the polymethyl methacrylate (PMMA) was coated on the graphene at 3000 RPM for 30 s. The PMMA/graphene/metal catalyst was separated from the Si substrate during floating on a buffered HF (BOE) for several minutes. The metal catalyst was then etched by floating on a 0.1 M ammonium persulfate solution. After rinsing the PMMA/graphene with DI-water several times, the PMMA/graphene was transferred onto the target substrate and baked at 180 °C for 30 minutes, increasing the adhesion between the graphene and target substrate. The PMMA was removed with acetone and IPA.

### Raman analysis

The Raman mapping images were obtained using a NTEGRA Spectra from NT-MDT, equipped with a thermoelectric (TE) cooled CCD and a ×50 objective lens (NA = 0.75). The excitation laser with a wavelength of 532 nm from a diode laser was used for all Raman measurements. The Si-, G-, and 2D-peak intensities were extracted from the full-spectrum mapping results and compared after normalization with the Si-peak intensity on the SiO_2_ substrate.

## Electronic supplementary material

Supplementary Information
