# Supplementary material for: Layer number identification of CVD-grown multilayer graphene using Si peak analysis
Source: Sci Rep. 2018 Jan 12;8:571. doi: 10.1038/s41598-017-19084-1 (PMC5766578; doi:10.1038/s41598-017-19084-1)
Supplement: Supplementary file 1 — Supplementary Information [file 41598_2017_19084_MOESM1_ESM.doc]

**Layer number identification of CVD-grown multilayer graphene using Si peak analysis**

You-Shin No,^1^ Hong Kyw Choi,^2^ Jin-Soo Kim,^3^ Hakseong Kim,^4^ Young-Jun Yu,^5^ Choon-Gi Choi,^6^ Jin Sik Choi^1^*

^1^Department of Physics, Konkuk University, Seoul 05029, Korea.

^2^Emerging Devices Research Group, Electronics and Telecommunications Research Institute (ETRI), Daejeon 34129, Korea.

^3^Department of Physics, Korea University, Seoul 02841, Korea.

^4^Korea Research Institute of Standards and Science (KRISS), Daejeon 34113, Korea.

^5^Department of Physics, Chungnam National University, Daejeon 34134, Korea.

^6^Graphene Research Lab., Emerging Devices Research Group, Electronics and Telecommunications Research Institute (ETRI), Daejeon 34129, Korea.

^*^E-mail: [jinschoi@konkuk.ac.kr](mailto:jinschoi@konkuk.ac.kr)


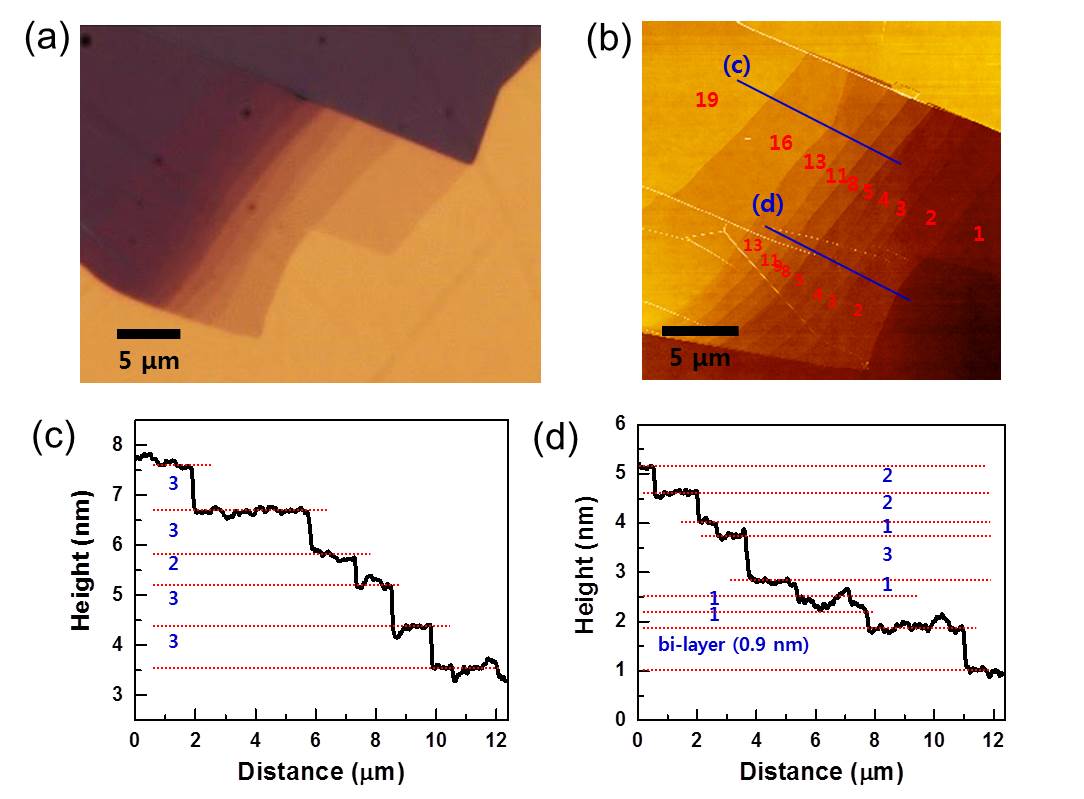


**Figure S1. Identification of the number of layers of mechanically exfoliated graphene.** (a) optical microscope image, (b) AFM topography, and (c) and (d) are the line profiles. The red dotted line indicates the plateau level, and a blue digit indicates the number of layers between the two red dotted lines.

Figure S1(a) shows an optical microscope image of the step-like featured multilayer graphene. We obtained the AFM topography using the tapping mode. The height profile shows that our graphene sample contained single- (1L), bi- (2L), tri- (3L), tetra- (4L), and penta- (5L) layers, and gradually increased in the layers with 2–3 layer spacing. Since the surface morphology of the SiO_2_ surface is relatively rough to accurately measure the height of the graphene layer, the layer spacing height was in the range of 0.28–0.37 nm. The spacing height of the 2-, and 3-layers exhibited 0.55–0.65 nm, and 0.88–0.93, respectively. By considering the ideal spacing distance of 0.33 nm, we distinguished the experimental results of the 1-, 2-, and 3-layered samples.


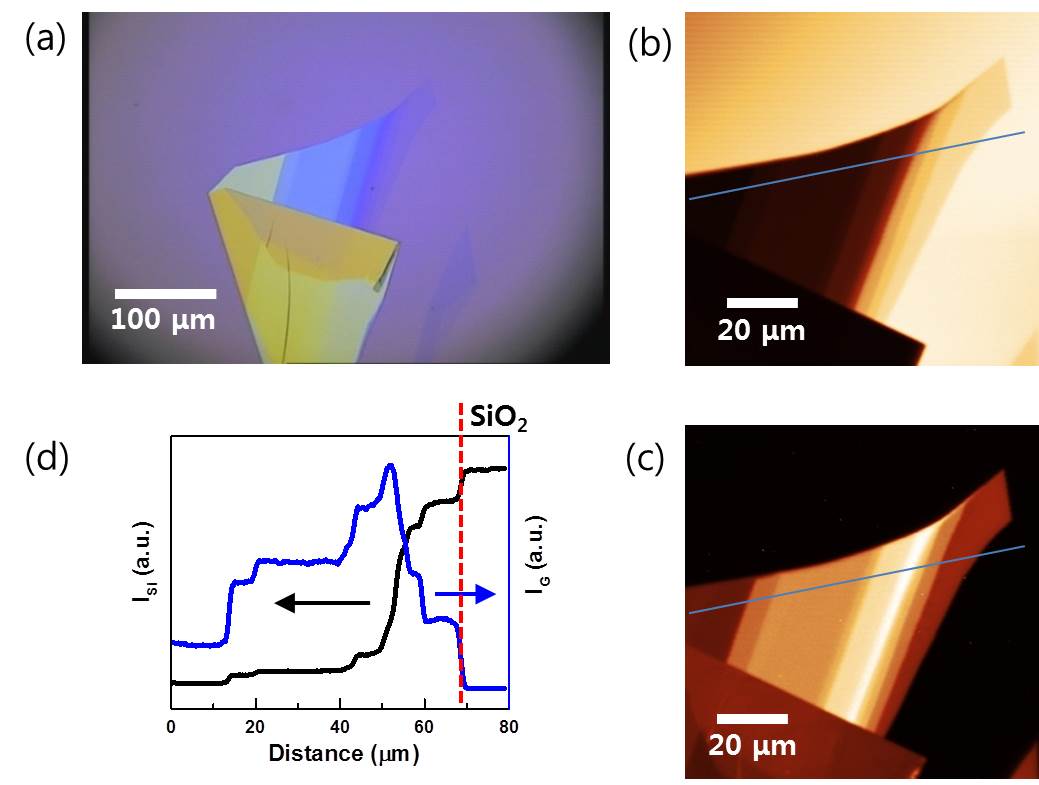


**Figure S2. Raman intensity mapping analysis of exfoliated multilayer graphene.** (a) Optical microscopy image of exfoliated multilayer graphene. Raman intensity mapping images of Si-peak (b) and G-peak (c). (d) Co-plotted Si-peak intensity (black line and arrow) and G-peak intensity (blue line and arrow) line profiles along the blue lines in (b) and (c).

We prepared another mechanically exfoliated multilayer graphene with a continuous increase of layer number, from single- to thick multi- layers. In the optical microscopy image, the color changes from blue to yellow as the number of graphene layers increases. This sample included thicker layers (yellow region) compared with the sample used in Figure 4 [optical microscopy image––Figure S1(a)]. We also observed a gradual intensity variation of the Si-peak as the number of layers increased or decreased in the whole region. We confirmed a significant decrease in the G-peak intensity of the exfoliated multilayer graphene in the thicker layer region (> 18 layers), but the Si-peak still monotonically decreased in this region.
